# Visfatin as a Novel Mediator Released by Inflamed Human Endothelial Cells

**DOI:** 10.1371/journal.pone.0078283

**Published:** 2013-10-10

**Authors:** Tania Romacho, Laura A. Villalobos, Elena Cercas, Raffaele Carraro, Carlos F. Sánchez-Ferrer, Concepción Peiró

**Affiliations:** 1 Departamento de Farmacología y Terapéutica, Universidad Autónoma de Madrid, Madrid, Spain; 2 Servicio de Endocrinología, Hospital Universitario de La Princesa and Departamento de Medicina, Universidad Autónoma de Madrid, Madrid, Spain; 3 Instituto de Investigación Sanitaria del Hospital de La Princesa, Madrid, Spain; Morehouse School of Medicine, United States of America

## Abstract

**Background:**

Visfatin is a multifaceted adipokine whose circulating levels are enhanced in different metabolic diseases. Extracellular visfatin can exert various deleterious effects on vascular cells, including inflammation and proliferation. Limited evidence exists, however, on the capacity of human vascular cells to synthesize and release visfatin by themselves, under basal or pro-inflammatory conditions.

**Methods and Results:**

Intracellular visfatin was detected by Western blot in non-stimulated human umbilical vein endothelial cells (HUVEC). However, exposing HUVEC for 18 h to a series of pro-inflammatory stimulus, such as interleukin (IL)-1β (1 to 10 ng/mL), tumor necrosis factor-α (1 to 10 ng/mL) or angiotensin II (10 pmol/L to 1 μmol/L) markedly enhanced intracellular visfatin content. Using IL-1β (10 ng/mL; 18 h), it was determined that the increase in intracellular visfatin, which was paralleled by enhanced visfatin mRNA levels, relied on a signalling mechanism involving both nuclear factor-κB and poly (ADP ribose) polymerase-1 activation. Moreover, IL-1β modified the sub-cellular localization of visfatin; while in non-stimulated HUVEC immunoreactive visfatin predominantly showed an intra-nuclear granular pattern, in IL-1β-inflamed cells an extra-nuclear filamentous staining, co-localising with F-actin fibers and suggesting a secretory pattern, was mainly found. Indeed, IL-1β promoted visfatin secretion, as determined by both ELISA and immunocytochemistry.

**Conclusions:**

Human endothelial cells synthesize and release visfatin, particularly in response to inflammation. We suggest that the inflamed endothelium can be a source of visfatin, which arises as a local inflammatory mediator and a potential therapeutic target to interfere with vascular inflammation.

## Introduction

Visfatin is a multifaceted molecule initially proposed to be mainly released by visceral fat [1], structurally identical to pre-B-cell colony-enhancing factor (PBEF) [2], and exhibiting nicotinamide phosphoribosyltransferase (Nampt) enzymatic activity [3,4]. For this reason, different reports have referred to this adipocytokine as PBEF/Nampt/visfatin [4,5]. Enhanced circulating levels of visfatin have been reported in patients affected by metabolic disorders, such as diabetes mellitus, obesity or the metabolic syndrome [6,7], which might be related to the development of cardiovascular complications linked to these diseases. Supporting this, plasma visfatin concentrations have been positively associated with vascular damage and endothelial dysfunction in type 2 diabetes mellitus [8] and chronic kidney disease [9]. Furthermore, enhanced visfatin content has been detected in human unstable atherosclerotic plaques [10], while it has been proposed as a novel marker of carotid atherosclerosis in type 2 diabetes [11].

There is growing evidence that extracellular visfatin can directly promote endothelial dysfunction by exerting a series of deleterious actions on the vascular wall [7,12]. In human vascular smooth muscle, visfatin can directly promote inflammation through the activation of the extracellular-signal regulated kinase (ERK) 1/2 - nuclear factor (NF)-κB - inducible nitric oxide synthase (iNOS) axis [5]. Moreover, visfatin derived from perivascular adipose tissue stimulates vascular smooth muscle cells proliferation [13]. In human endothelial cells, visfatin promotes NF-κB activation, leading to the expression of vascular adhesion molecules, matrix metalloproteinases activation and the release of cytokines and chemokines, including interleukin (IL)-6 or monocyte chemotactic protein-1 [14–16]. Moreover, while a vasculoprotective role of visfatin has been claimed through the release of endothelial nitric oxide [17], other studies have shown the capacity of visfatin to impair endothelium-dependent relaxation in isolated mesenteric microvessels from both animals and humans [18,19]. Some of the deleterious vascular effects elicited by visfatin seem to rely on Nampt enzymatic activity, which transforms nicotinamide into nicotinamide monucleotide [5,13,19].

Despite the interest in understanding the vascular impact of exogenous visfatin, little is known about the capacity of vascular cells themselves to synthesize and release visfatin that might act as an autocrine or paracrine regulator within the vasculature. In the present work, we have explored such capacity using human endothelial cell cultures, with special attention to the role played by inflammation, considered as a hallmark of atherothrombotic diseases.

## Materials and Methods

### Ethics Statement

The investigation conforms to the principles outlined in the Declaration of Helsinki and to Spanish legal dispositions. Experiments with human umbilical vein endothelial cells were reviewed and approved by the ethics committee of Universidad Autónoma de Madrid and Hospital Universitario de Getafe. A written informed consent was obtained from every umbilical cord donor. 

### Materials

Culture plasticware was from TPP (Tragadingen, Switzerland). M199 medium and fetal calf serum (FCS) was from Biological Industries (Beit-Haemek, Israel). IL-1β with an endotoxin level below 0.1 ng per μg, was from Peprotech (London, UK). Endothelial cell growth supplement (ECGS), pyrrolidine dithiocarbamate (PDTC), PJ34 and, unless otherwise stated, all other reagents were from Sigma Chemical Co. (St. Louis, MO, USA).

### Cell culture

Human umbilical vein endothelial cells (HUVEC) were enzymatically isolated, as previously described [20]. For experiments, cells at passages 1-4 were incubated with different compounds in M199 medium supplemented with 10% FCS, ECGS and antibiotics for 18 h. The investigation conforms to the principles outlined in the Declaration of Helsinki. Experiments with human cells were reviewed and approved by the ethics committee of Universidad Autónoma of Madrid and Hospital Universitario of Getafe, respectively, and written informed consent was obtained from all donors.

### Western blotting

Proteins (20 μg) were separated by SDS-PAGE, transferred to nitrocellulose membranes (Whatman, Kent, UK) and probed with a primary polyclonal antibody against either PBEF/Nampt/visfatin (dilution 1/500; Affinity Bioreagents, Golden, CO, USA) or PARP-1 (dilution 1/1000, Trevigen, Gaithersburg, MD, USA), followed by an appropriate horseradish peroxidase-conjugated secondary antibody (dilution 1/10,000; Millipore, Bedford, MA, USA), as previously described [5]. Immunoreactive bands were detected by enhanced chemiluminescence (GE Healthcare, Uppsala, Sweden) and quantified using NIH Image software. The membranes were probed with an anti α-tubulin primary antibody (dilution 1/10,000) to ensure equal loading.

### Indirect immunofluorescence

Visfatin was localized in HUVEC by indirect immunofluorescence using a primary polyclonal antibody against visfatin (dilution 1/50, Affinity Bioreagents, Golden, CO, USA), followed by a FITC-conjugated secondary antibody (dilution 1/200). To visualize F-actin filaments, cells were co-stained with phalloidine-TRITC (dilution 1/200). Cell nuclei were counterstained with 1 μmol/L 4'-6-diamidino-2-phenylindole (DAPI; Molecular Probes-Invitrogen Corporation, Carlsbad, CA, USA) and observed under an Eclipse TE300 epifluorescence microscope (Nikon, Tokyo, Japan) and a spectral confocal microscope (LEICA TCSSP5-AOBS, Leica microsystems, Heidelberg, GMBH, Germany). Confocal images were analysed with LAS AF software, version 1.5.1 Build 869 (LEICA).

### Reverse-transcriptase polymerase chain reaction (RT-PCR)

Total RNA was extracted using a commercial kit (RNeasy, Qiagen; Hilden, Germany), quantified by measuring absorbance at 260 nm and aliquots of 1μg RNA were reverse-transcribed using a commercial kit (iScript CDNA Synthesis kit, Bio-Rad, Hercules, CA, USA). The resulting cDNA was amplified using human visfatin primers (5’GGAGGGTGACGGGGTGAAGG3’, 5’GTCGGTGGCCAGGAGGATGTT3’) and human 18S as an internal control (5’GGAAGGGCACCACCAGGAGT3’, 5’TGCAGCCCCGGACATCTAAG3’). The reaction was conducted in an ATC 401 thermocycler (Nyx Technik, San Diego, CA, USA), with an initial denaturation step at 95°C for 3 min, followed by 30 cycles each consisting of incubation at 94°C for 30s, 59°C for 40s, and 72°C for 30s. Aliquots of the resulting PCR products were loaded on 1% agarose gels containing ethidium bromide. The resulting bands were visualized under ultraviolet light in a transiluminator coupled to a camera (Chemidoc™ XRS System, Bio-Rad, Hercules, California, USA), and quantified using NIH Image software (Image J version 1.4).

### Detection and quantification of visfatin secretion

Visfatin secretion by endothelial cells was visualized by adapting a previously described immunohistochemistry protocol [21] to HUVEC seeded on Immobilon-P membranes (Millipore, Bedford, MS, USA) coated with type I collagen. With this procedure the cell secretion products become firmly bound to the membrane. At the end of the treatments, the membranes were collected, fixed with Bouin’s fixative and blocked. Endogenous peroxidase was inhibited with 1% hydrogen peroxide and the membranes were probed with an antibody against human visfatin (1/50), followed by HRP-conjugated secondary antibody and addition of a diaminobencidine-based substrate for peroxidase (Vector Laboratories, Burlingame, CA, USA). Air-dried membranes mounted on coverslips were visualized on a brightfield microscope Eclipse TE300 (Nikon, Tokio, Japan). Images were captured with a SPOT 1.3.0 camera coupled to the microscope with Adobe Photoshop 6.0. software. In parallel experiments, visfatin was quantified in cell supernatants with an extracellular visfatin detection kit (Adipogen, Incheon, South Korea) following the manufacturer’s guidelines. The absorbance at 450 nm was measured in an ELISA plate reader ELx 800 (BIO-TEK Instruments, Winooski, VT, USA).

### Statistical analysis

Results are expressed as mean ± SEM of at least three independent experiments. Statistical analysis was performed using ANOVA followed by Fisher’s LSD test for curves or Student’s t-test for data points, with the level of significance chosen at *P*<0.05.

## Results

### Inflammation promotes visfatin synthesis in HUVEC

**Figure 1:**
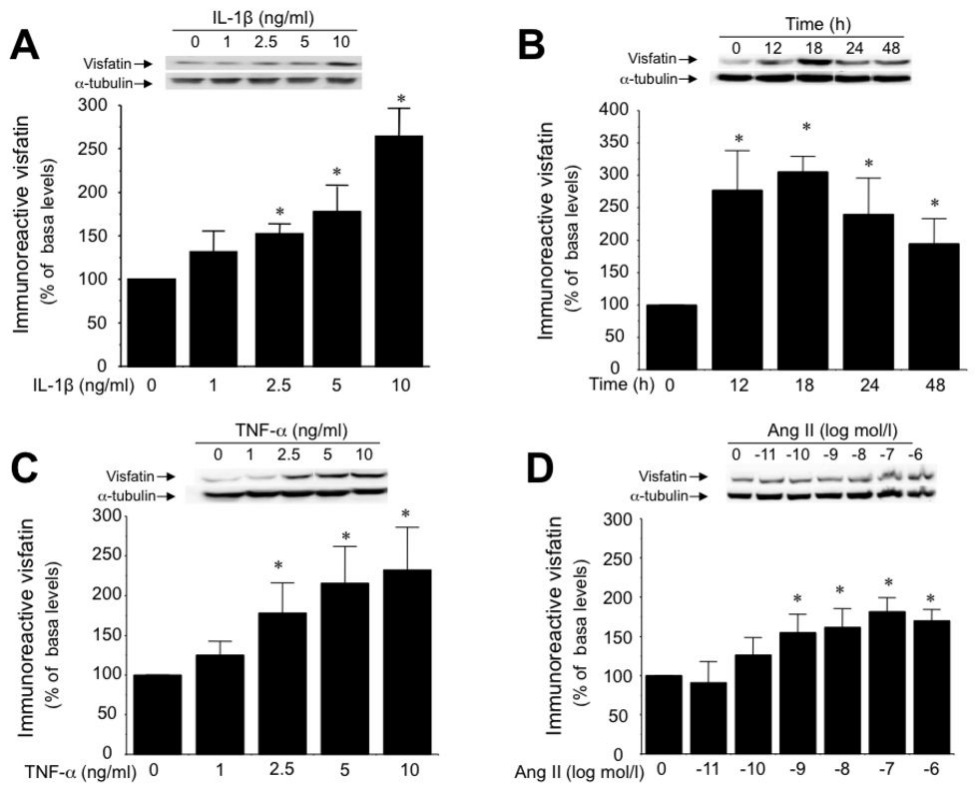
Inflammation enhances intracellular visfatin levels in HUVEC. (A) Concentration-dependent effect of IL-1β (1 to 10 ng/mL; 18 h) on cellular visfatin content determined by Western blotting. (B) Time course of visfatin induction by IL-1β (10 ng/mL) over 48 h. Visfatin content was also determined in HUVEC challenged for 18 h with either (C) TNF-α (1 to 10 ng/mL) or (D) Ang II (10 pmol/L to 1 µmol/L). Data are the mean±SEM of five independent experiments. *P<0.05 vs non-stimulated cells. Representative gels are shown on the top.

Using Western blot techniques, immunoreactive visfatin was detected in HUVEC cultures under non-inflammatory conditions (Figure 1A). However, after challenging the cells with a well-known pro-inflammatory stimulus, such as the cytokine IL-1β (1 to 10 ng/mL; 18 h), a concentration-dependent increase in the cellular levels of visfatin was observed, with a threshold at 2,5 ng/mL (Figure 1A). At the 10 ng/mL concentration, IL-1β already significantly increased visfatin levels at 12 h, with a maximum reached at 18 h, then followed by a slow and progressive decay at 24 and 48 h (Figure 1B). Moreover, the stimulation of HUVEC with two other vascular pro-inflammatory stimuli such as tumor necrosis factor (TNF)-α (1 to 10 ng/mL; 18 h) or angiotensin II (10 pmol/L to 100 nmol/L; 18h) also resulted in a concentration-dependent enhancement of cellular visfatin content at thresholds of 2.5 ng/mL and 1 nmol/l, respectively (Figures 1C and 1D).

**Figure 2:**
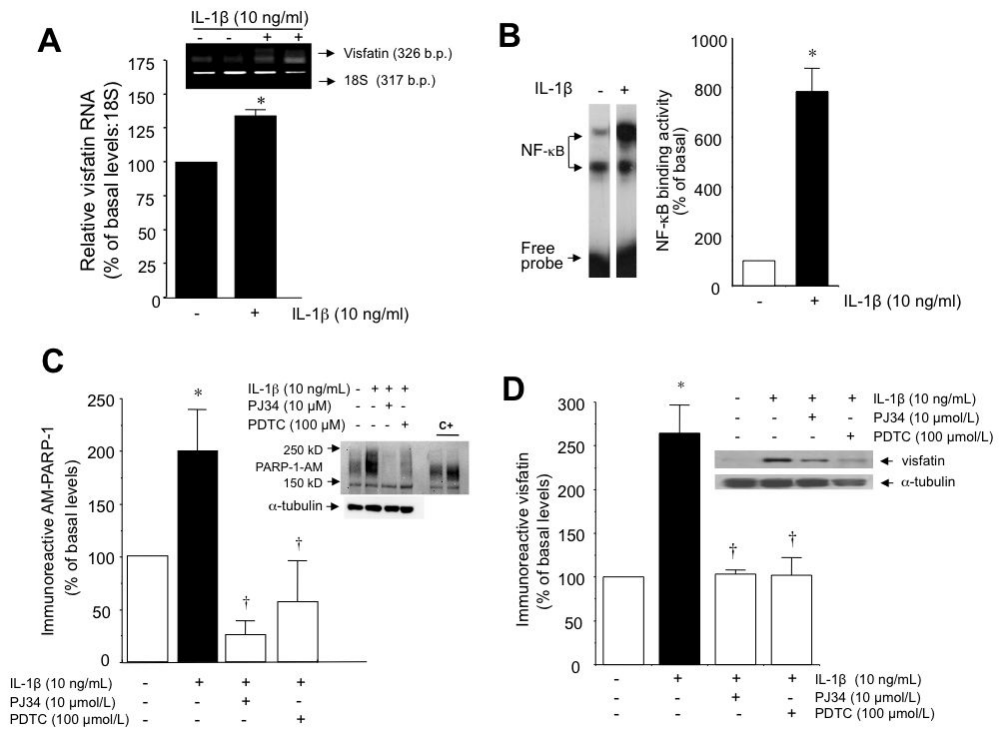
The sequential activation of NF-κB and PARP-1 activation mediates inflammation-evoked visfatin production. (A) Relative mRNA expression of visfatin in HUVEC treated with or without IL-1β (10 ng/mL; 18 h). (B) NF-κB activation in HUVEC after incubation with IL-1β (10 ng/mL) for 1 h was determined by EMSA. Data are the mean±SEM of four independent experiments. *P<0.05 vs basal levels. (C) Auto-modified immunoreactive PARP-1 in HUVEC stimulated for 18 with or without IL-1β (10 ng/mL; 18 h), in the absence or presence of the respective PARP-1 and NF-κB inhibitors PJ34 (10 µmol/L) and PDTC (100 µmol/L). Data are the mean±SEM of four independent experiments. *P<0.05 vs basal; †*P*<0.05 vs IL-1β. (D) Visfatin protein levels in HUVEC treated with IL-1β (10 µmol/L; 18 h) with or without PJ34 (10 µmol/L) or PDTC (100 µmol/L). Data are the mean±SEM of four independent experiments. *P<0.05 vs basal; †*P*<0.05 vs IL-1β. Representative gels are shown.

Furthermore, IL-1β (10 ng/mL; 18 h), selected as a model of pro-inflammatory stimulus, increased HUVEC visfatin mRNA levels, suggesting a *de novo* synthesis of visfatin triggered by the cytokine (Figure 2A).

### Nuclear factor-κB and poly (ADP ribose) polymerase-1 mediate visfatin synthesis by HUVEC in response to inflammation

To gain insight into the intracellular pathways mediating the induction of visfatin by inflammation in HUVEC, the implication of nuclear factor (NF)-κB and poly (ADP ribose) polymerase (PARP)-1 activation, as relevant molecules involved in inflammatory responses, was next explored. Figures 2B and 2C illustrate the stimulation of NF-κB DNA binding activity and PARP-1 activity by IL-1β (10 ng/mL; 18 h). respectively. Indeed, a sequential NF-κB - PARP-1 activation pathway was indicated by the fact that the NF-κB inhibitor PDTC (10 µmol/L) abrogated PARP-1 activation by IL-1β (Figure 2C). Both NF-κB and PARP-1 were necessary for visfatin induction in HUVEC, as their respective inhibitors PDTC and PJ34 (100 µmol/L) prevented the increase in visfatin levels triggered by IL-1β (Figure 2D).

### Inflammation modifies the sub-cellular distribution of visfatin in HUVEC

**Figure 3:**
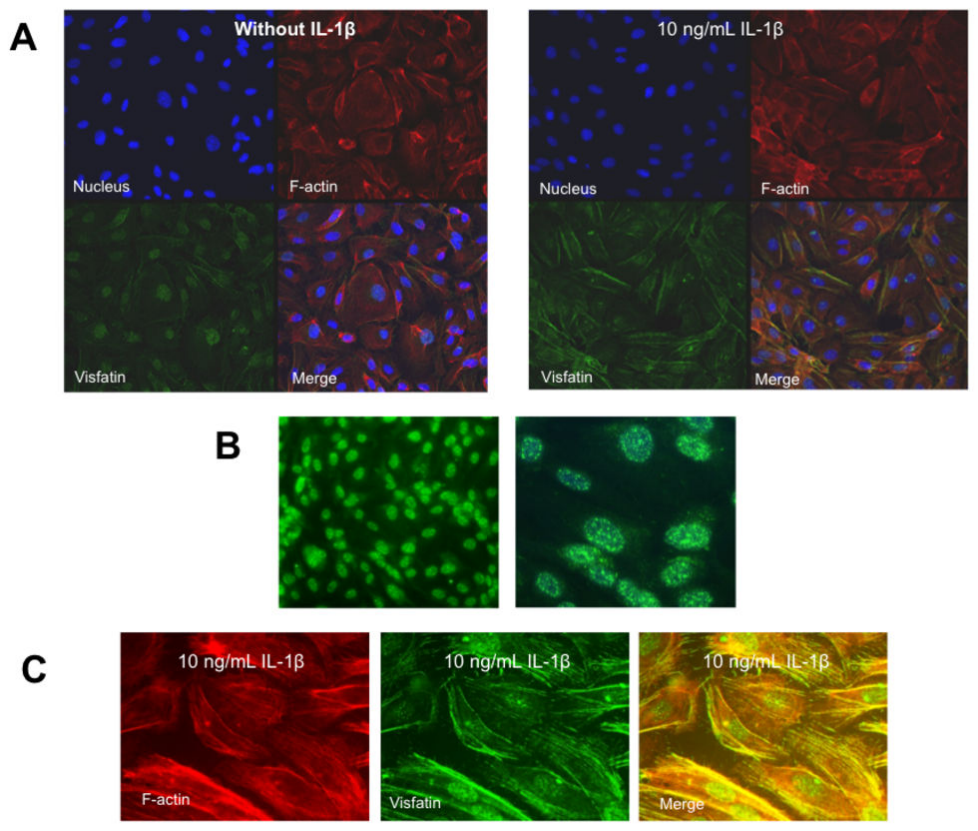
Inflammation modifies the sub-cellular distribution pattern of visfatin in HUVEC. (A) Confocal maximum projections showing a general view of stained nuclei (blue), visfatin (green), F-actin (red), and merge (yellow) in HUVEC cultures. In non-stimulated HUVEC (left), visfatin is mainly localized within the cell nucleus, while in HUVEC stimulated with IL-1β (10 ng/mL) for 18 h (right), visfatin can be markedly found in non-nuclear localizations together with F-actin. 630x magnification. (B) Magnified view of the visfatin granular nuclear distribution in non-stimulated HUVEC at 400x (left) and 1,000x (right) magnification. Nuclei were counterstained with 4'-6-diamidino-2-phenylindole (DAPI). (C) Magnified view of the visfatin non-nuclear filamentous distribution pattern in HUVEC stimulated with IL-1β (10 ng/mL). 1,000x magnification.

Using immunofluorescence techniques, we observed that, under non-inflammatory conditions, visfatin predominantly exhibited a granular pattern localized within the cell nucleus (Figures 3A, left panel, and Figure 3B). However, 18 h after exposing HUVEC to IL-1β (10 ng/mL) a marked positive extra-nuclear filamentous staining for visfatin was also observed (Figure 3A right panel and Figure 3C). To analyse whether these filaments did co-localize with some cytoskeleton element, we used fluorescence-labelled phalloidin to detect F-actin. Figures 3A and 3C show that IL-1β promoted the co-localization of visfatin with F-actine fibers, particularly in the cytoplasm and at the cell surface (Figure 3A right pannel and 3C), suggesting a secretory pattern. 

### Inflammation promotes visfatin secretion by HUVEC

**Figure 4:**
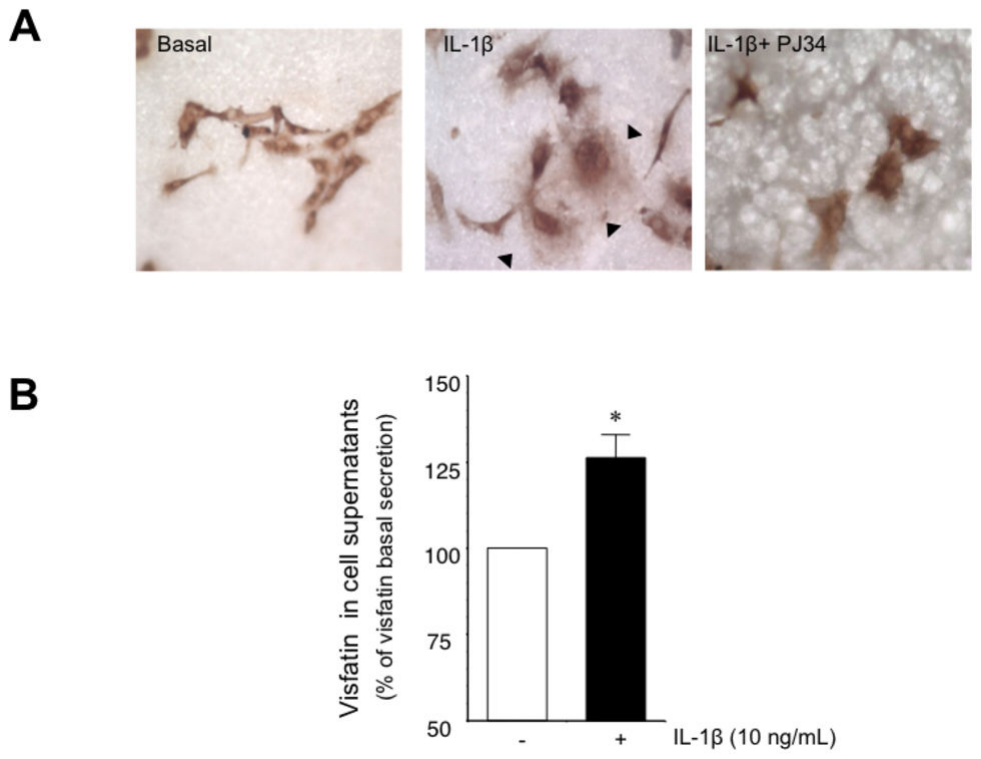
Inflammation promotes visfatin secretion by HUVEC. (A) Representative microphotographs of visfatin release from HUVEC grown on Immobilon-P membranes and treated with or without IL-1β, (10 ng/mL; 18 h) in the absence or the presence of the PARP-1 inhibitor PJ34 (10 µmol/L). Secreted visfatin appears as a diffuse halo of extracellular positive immunostaining (black arrows). Magnification 400x. (B) Visfatin content determined by ELISA in cell supernanants treated with or without IL-1β (10 ng/mL; 18 h). Data are the mean±SEM for four independent experiments. *P<0.05 vs basal.

There has been controversy about the capacity of visfatin to be secreted to the extracellular space, as this protein lacks a signal peptide [22,23]. To explore the capacity of HUVEC to secrete visfatin, we first used an immunocytochemical approach to detect visfatin in the extracellular space [21]. As shown in Figure 4A, IL-1β promoted visfatin release, visualized as a stained halo surrounding each secreting cell. This secretion halo could not be observed in HUVEC treated with the PARP-1 inhibitor PJ34 (100 µmol/L; Figure 4A). As a second approach, visfatin was quantified using ELISA, and significantly higher levels were found in the supernatant of HUVEC challenged with IL-1β as compared with non-stimulated ones (Figure 4B).

## Discussion

A few years ago, Fukuhara et al [1] first identified visfatin as a new adipocytokine identical to PBEF, a 52 kDa cytokine acting on early B-lineage precursor cells [2], and to the enzyme Nampt [3,4], which plays an essential role in the biosynthesis of NAD^+^ by converting nicotinamide into nicotinamide mononucleotide (NMN), which is then transformed into NAD^+^ by nicotinamide/nicotinic acid mononucleotide adenyltransferase (Nmnat) [24]. Despite in the last years different studies have demonstrated the capacity of exogenous visfatin to directly induce vascular cell damage [5,13–16,19], whether vascular cells themselves synthesize and release visfatin to the extracellular milieu has been only scarcely addressed.

In the present study, immunoreactive visfatin was detected in cultured human endothelial cells. Most noticeably, the intracellular visfatin levels were markedly enhanced in response to a series of molecules implicated in vascular inflammation and disease, such as IL-1β, TNF-α or Ang II. Therefore, one first finding of this study was that inflamed human endothelial cells over-produce visfatin. This is most likely explained by a *de novo* visfatin synthesis, as indicated by the increase of visfatin mRNA levels in response to a pro-inflammatory stimulus.

Supporting this observation, Williams et al [25] showed in a wide microarray study that visfatin was among the factors whose mRNA was increased upon IL-1β -stimulation in HUVEC, although the post-transcriptional impact of such finding on visfatin protein levels was not explored. Other reports using different cell types have reported visfatin production in response to inflammatory conditions such as labour, acute lung injury, sepsis or rheumatoid arthritis [26–29]. Moreover, here we have gained insight into the signalling mechanisms mediating visfatin induction by inflamed endothelial cells by identifying the sequential activation of NF-κB and PARP-1 as a key event in such process.

A second main finding of the present study was that inflammation not only increased visfatin synthesis but also modified the sub-cellular distribution of visfatin while enhancing the secretion of the adipokine to the extracellular space. In line with this observation, changes in the sub-cellular visfatin distribution have been previously reported in non-proliferating PC-12 cells or confluent Swiss 3T3 fibroblasts when these cell types are activated by proliferative stimuli [22]. Indeed, two different forms of Nampt/visfatin, both intracellular and extracellular, have been identified to date. On one hand, the intracellular form would play a central role in maintaining the activity of different NAD-dependent enzymes that are implicated in the regulation of cell metabolism [4,24]. This form has been involved as a NAD supplier for a number of NAD-consuming enzymes acting intra-nuclearly, which are involved in key enzymatic reactions for cell growth and survival [30,31]. In human vascular smooth muscle cells, the intracellular form has been identified as a regulator of NAD-dependent protein deacetylase activity, promoting cell maturation and increasing lifespan [32,33], while improving the functionality and angiogenic capacity, as well as its replicative lifespan, in human endothelial cells [34,35]. In this context, the present study localizes intra-nuclear Nampt/visfatin as the predominant form in non-inflamed endothelial cells, which most likely reflects cell maintenance functions.

On the other hand, the extracellular form of Nampt/visfatin is synthesized and released to the extracellular milieu, where it could exert a variety of actions in a paracrine or endocrine manner [4]. Structurally, extracellular visfatin shows a slightly higher molecular weight than the intracellular isoform and seems to undergo post-transcriptional modifications [4,24]. Here, we have demonstrated the secretory activity of human endothelial cells through both the immunolocalization and quantification of visfatin in the extracellular space, which was particularly evident under inflammatory conditions. There has been controversy on the ability of visfatin to be secreted to the extracellular milieu, since this protein lacks a signal sequence for secretion [3,22,23]. However, other well-characterised peptides lacking a signal sequence for secretion, including the pro-inflammatory cytokine IL-1β itself, are released by a wide variety of cell types [36,37]. 

In the last years, a role for visfatin as a possible link between metabolic disorders and atherothrombotic inflammatory diseases has been supported [38]. Enhanced circulating visfatin has been proposed as an atherosclerosis marker [11,39], while other studies suggest that it rather reflects the global inflammatory status in patients with cardiovascular and renal diseases [40]. Not only circulating visfatin, but also perivascular adipose tissue-derived visfatin has been related to coronary and aortic atherosclerosis [41]. This observation highlights that locally produced visfatin may play an important paracrine role in the development of atherosclerotic lesions. In this context, activated monocytes/macrophages that closely interact with vascular cells do release visfatin [42]. Increased visfatin expression has been described in macrophages of human unstable carotid and coronary atherosclerotic plaques [10] suggesting that locally produced visfatin should be regarded as an inflammatory mediator with a role in plaque destabilization. In the present study, we have demonstrated that human endothelial cells, which constitutively express visfatin, synthesize and release significantly higher amounts of the adipokine in response to inflammation. Therefore, not only macrophages and perivascular adipose tissue, but also the inflamed endothelium itself represents a local source of visfatin that may promote and amplify vascular damage. 

In conclusion, human endothelial cells synthesize and secrete visfatin, which is particularly reinforced in a pro-inflammatory environment. Whilst acknowledging the limitations of an *in vitro* study, we propose that visfatin released by endothelial cells may act as a local pro-inflammatory mediator in the vascular wall with a potential role in atherothrombotic diseases.
